# Supplementary material for: General psychiatric inpatient care for persons with anorexia nervosa—an integrative literature review protocol
Source: BMJ Open. 2023 Sep 29;13(9):e076437. doi: 10.1136/bmjopen-2023-076437 (PMC10546143; doi:10.1136/bmjopen-2023-076437)
Supplement: Author's manuscript [file bmjopen-2023-076437.draft_revisions.pdf]

BMJ Open

BMJ Open is committed to open peer review. As part of this commitment we make the peer review history of every article we publish publicly available.

When an article is published we post the peer reviewers' comments and the authors' responses online. We also post the versions of the paper that were used during peer review. These are the versions that the peer review comments apply to.

The versions of the paper that follow are the versions that were submitted during the peer review process. They are not the versions of record or the final published versions. They should not be cited or distributed as the published version of this manuscript.

BMJ Open is an open access journal and the full, final, typeset and author-corrected version of record of the manuscript is available on our site with no access controls, subscription charges or pay-per-view fees (http://bmjopen.bmj.com).

If you have any questions on BMJ Open's open peer review process please email info.bmjopen@bmj.com

BMJ Open

General psychiatric inpatient care for persona with anorexia nervosa - an integrative literature review protocol

Journal:	*BMJ Open*
Manuscript ID	bmjopen-2023-076437
Article Type:	Protocol
Date Submitted by the Author:	08-Jun-2023
Complete List of Authors:	Sandsten, Anna; Umea universitet Medicinska fakulteten, Nursing<br>Lindgren, Britt-Marie; Umeå universitet Medicinska fakulteten, Nursing<br>Strömbäck, Maria; Umea University Department of Community Medicine and Rehabilitation, Nursing<br>Ejneborn-Looi, Git-Marie; Luleå University of Technology, Department of Health Sciences<br>Gabrielsson, Sebastian; Luleå University of Technology, Department of Health Sciences
Keywords:	Eating disorders < PSYCHIATRY, Hospitalization, PSYCHIATRY, MENTAL HEALTH

SCHOLARONE™
Manuscripts

General psychiatric inpatient care for persons with anorexia nervosa – an integrative literature review protocol

Anna Sandsten, PhD Student, MSc, Specialist Nurse in Psychiatric Care, Department of Nursing, Umeå Center for Gender Studies, Gender Research School, Umeå University. E-mail: anna.sandsten@umu.se

Britt-Marie Lindgren, PhD, Associated Professor, Specialist Nurse in Psychiatric Care, Department of Nursing, Umeå University. E-mail: britt-marie.lindgren@umu.se. Orcid nr 0000-0002-3360 5589

Maria Strömbäck, PhD, Senior Lecturer, Specialist Physiotherapist, Department of Community Medicine and Rehabilitation, Umeå University. E-mail: maria.stromback@umu.se

Git-Marie Ejneborn Looi, PhD, Senior Lecturer, Specialist Nurse in Psychiatric Care, Department of Health, Learning and Technology, Luleå University of Technology. E-mail: git-marie.ejneborn-looi@ltu.se

Sebastian Gabrielsson, PhD, Associate Professor, Specialist Nurse in Psychiatric Care, Department of Health, Learning and Technology, Luleå University of Technology. E-mail: sebastian.gabrielsson@ltu.se Orcid nr 0000-0002-1624.1795

Corresponding author Anna Sandsten, e-mail: anna.sandsten@gmail.com, +46702349993.

Author contributions All authors contributed to the conception of the review protocol and the study design of the review. AS and SG developed the search strategy together with a librarian and prepared supplementary materials. AS lead the writing of the manuscript, incorporated feedback from the other authors through multiple drafts. AS and SG prepared correspondence with the publisher. All authors contributed to the final manuscript and agreed with its publication.

ABSTRACT

Introduction People diagnosed with anorexia nervosa are offered or compelled to undergo various treatments and therapies. In Sweden, the lack of highly specialized eating disorder services means that people with anorexia nervosa might be cared for in general psychiatric wards. However, there has been limited research attention paid to how this specific context might benefit or harm persons with anorexia nervosa. There is also limited evidence regarding which form of care is most effective in anorexia nervosa and which type of treatment is optimal in general psychiatric inpatient care. Our intention is to highlight personal and professional perspectives of care for patients with anorexia nervosa in this context, with a focus on person-centred care, gender, and power. This protocol describes an integrative literature review aiming to synthesize existing knowledge on general psychiatric inpatient care for persons with anorexia nervosa.

Methods and analysis The study will be an integrative literature review following Whittemore and Knafl. We will search the academic databases PubMed, CINAHL, and PsycInfo in accordance with a specific search strategy determined in collaboration with a librarian with expertise on systematic reviews. Data will be analysed using a constant comparison method. Persons with their own experience of anorexia nervosa in general psychiatric inpatient care will be involved as co-researchers.

Ethics and dissemination As this is a literature review, formal ethical review is not required. The findings will be disseminated through peer-reviewed publication and presented at conferences related to this field.

Amendments If we need to amend this protocol, we will give the date of each amendment, describe the change, and give the rationale in this section.

Sponsor and founding This research received no specific grant from any funding agency in the public, commercial, or non-profit sectors.

For peer review only - http://bmjopen.bmj.com/site/about/guidelines.xhtml

PROSPERO registration number CRD42023426095

STRENGTHS AND LIMITATIONS OF THIS STUDY

- This review will provide knowledge of the content of nursing in general psychiatric inpatient care for persons with anorexia nervosa.
- Persons with their own experiences of anorexia nervosa in general inpatient care will contribute their knowledge and experience as co-researchers and through reading and commenting on the article.
- The publication of this protocol ensures transparency, and evaluation by independent reviewers of all studies during screening and selection reduces the risk of bias in the selection of included studies.
- An integrative literature review combines findings from quantitative and qualitative research which ensures full coverage of research knowledge in the field.
- A limitation is that we exclude studies in languages other than Swedish and English which may lead to potentially relevant studies not being included.

INTRODUCTION

Anorexia nervosa (AN) is reported worldwide among people of all genders and ages. The incidence among younger persons (aged <15) has increased in recent decades, but the reason for this remains unclear and requires further research (1). Quality of life for those having AN can be very low, and severe long-term AN significantly increases the risk of premature death due to physical stress or suicide (2). The psychological foundation of AN can be described by feelings of self-criticism, low self-esteem, isolation, captivity in the disease, and hopelessness about the possibility of a good life (3). On the one hand, AN is a psychiatric diagnosis with a large negative impact on social ability (4), but on the other, it can be experienced as psychologically meaningful. For example, strict rules regarding diet and activity can create structure and predictability in everyday life. Furthermore, AN can function as a strategy to avoid difficult feelings, and can contribute to the experience of control and inner strength (5,6).

Persons who can no longer manage to cope with their psychiatric illness at home are able to receive psychiatric inpatient care at a hospital (7). However, the lack of highly specialized eating disorder services means that persons with AN might be cared for in general psychiatric wards, which constitute an environment characterized by rules, locked doors, and a lack of choice (8). This can be stressful, but at the same time being in a hospital can allow feelings of being safe. General psychiatric inpatient care can be voluntary or coercive, and persons experiencing AN can be offered or compelled to undergo various treatments and therapies (7).

General psychiatric inpatient care in psychiatry typically means that persons with different diagnoses are cared for in the same place. This leads to a mix of persons with different needs (8) - for example, a person experiencing a manic episode may need reduced stimuli at the same time as a person with depression may need support in finding meaningful activities, and a person with AN may need support in reducing their activity level. Staff describe difficulties in switching between patients' different needs (9), and wish for strategies and resources for a meaningful care (10). General psychiatric inpatient care can be understood as a closed system which has specific ideas and expectations of both staff and patients (11). Staff describe difficulties in maintaining relationships with persons with AN who experience positive aspects of the disease, leading to ambivalent feelings towards care (12,13). Perceiving a

For peer review only - http://bmjopen.bmj.com/site/about/guidelines.xhtml

person as "difficult to treat" can inspire feelings of insecurity in staff. Uncertainty may lead to an increased tendency to rely on guidelines in the care of persons with AN, rather than finding individualized solutions (14).

According to the National Institute for Health and Care Excellence (15), treatment for AN should focus on psychoeducation, monitoring of weight, mental and physical health, and risk factors. Care should also be multidisciplinary and involve family members. These recommendations highlight that helping persons with AN to reach a healthy body weight is a key goal, and that weight gain is key in supporting other psychological, physical, and quality of life changes that are needed for improvement or recovery. In person-centred care, it is important to empower the patient to have an active role in their own care, to create good relationships, and to see the whole person beyond the disease (16). In recovery-oriented care, the patient owns their narratives in order to create security and enable them to use dialogue to express their expectations and wishes regarding the care and the goals of the care (11). To enable a turning point towards recovery for persons with AN, it is of great importance for the care to focus on both physical and psychological well-being (17). Personal recovery from AN is characterized by being seen and understood as an individual, by receiving support in finding meaning in life beyond illness, by feeling hope for recovery, and by regaining control and assuming responsibility over life (18).

Research indicates that care is influenced by societal gender norms, which in turn affect how healthcare professionals interact with patients and interpret patient complaints. Consequently, these gendered expectations may result in patients receiving varying examinations, treatments, and medications without sufficient medical justification (19). This study will summarize the care provided to individuals with AN within the setting of general psychiatric inpatient care. Emphasis will be placed on examining the content of care and the outcomes of care, exploring the discourse surrounding the diagnosis, and of the persons having the diagnose. Additionally, the study will shed light on gendered expectations and power imbalances that exist in this particular context.

For peer review only

Aim

The aim of this study is to synthesize existing knowledge on general psychiatric inpatient care for persons with AN.

Research questions

The review will seek to answer the following research questions:

- How is the content of care described?
- What is the focus of these descriptions? Opportunities/limitations?
- What outcomes are described?
- How are persons experiencing AN described?
- How is AN described?
- Which factors influence care for persons with AN?
- Are gender or power perspectives included in studies?
- How is person-centred care described?

For peer review only - http://bmjopen.bmj.com/site/about/guidelines.xhtml

METHODS AND ANALYSIS

Study design

This will be an integrative literature review conducted according to the method of Whittemore and Knafl´s (20). The purpose of using this method is to cover a large number of studies and to avoid excluding any research on the basis of method.

Inclusion and exclusion criteria

The studies included in the review will be restricted to those where the setting is general psychiatric inpatient care. Studies describing general psychiatric care for persons of all ages with AN will be included. We will also include studies that describe interventions in general psychiatric inpatient care for persons with AN, and that have a patient, relative, and/or staff perspective. Only studies that are peer-reviewed and published in scientific journals will be included. Language of publication will be restricted to English and Swedish, due to the authors` knowledge of languages. Studies with a focus on eating disorders in general will be excluded.

Information sources

The literature search will start in the spring of 2023, and we will aim for the study to be finished in January of 2024. Based on the specific purpose of the study and its inclusion and exclusion criteria, we will systematically search for knowledge and understanding in studies published in academic databases (20,21). We will use medical subject headings (MeSH) (22), CINAHL headings, and text words related to AN and inpatient care. Databases used in the search will be PubMed, CINAHL, and PsycINFO. We will also hand-search the reference lists of research and review articles to make sure that all relevant material has been captured.

Search strategy

The key concepts in this study are anorexia nervosa and inpatient care, and so the search terms will be "anorexia nervosa", "anorexia", "inpatients", "inpatient care", and "hospitalization". These search terms are combined using the Boolean operators AND and OR (22). A block search will be performed with search terms in the form of MeSH-terms (22), CINAHL headings, and free text. The literature search will be limited to peer-reviewed articles published in English. No study design or date limits will be imposed on the search, and similar searches will be performed in all databases (Table 1). Any duplicates will be removed. A PRISMA flow diagram (Figure 1) will be used to organize and summarize the search process (23). Finally, we will circulate a bibliography of the included articles to the systematic review team. The literature search strategy has been developed in collaboration with a librarian with expertise in systematic reviews at Luleå University of Technology.

For peer review only - http://bmjopen.bmj.com/site/about/guidelines.xhtml

Table 1	Draft PubMed search		
**Database: PubMed Date: 03-07-23**	Search terms	Limitations	Results
**#1**	anorexia nervosa [MeSH Terms] OR anorexia [Title/Abstract]		36 701
**#2**	inpatients [MeSH Terms] OR hospitalization [MeSH Terms] OR inpatient care [Title/Abstract]		312 995
**#3**	#1 AND #2	Full text, English, Swedish	991

Data management and quality appraisal

The literature search results will be uploaded to Rayyan, an Internet-based software program that can be used to facilitate collaboration among reviewers during the process of selecting studies (24). To appraise the methodological quality of studies with different types of methods (i.e. qualitative, quantitative, and mixed-methods studies), the Mixed Methods Appraisal Tool (MMAT) (25) will be used. The MMAT guides users through four steps: 1) the screening questions are applied to all studies. 2) for each relevant study, the type of design is determined and the corresponding criteria are used to appraise the study's quality, 3) two independent reviewers conduct the appraisal process and 4) an overall quality score is determined for each study (25). Because of the aim of this review, no studies will be excluded due to quality instead quality will be visualized and reflected on in the analysis section.

Selection process

Two authors will independently examine the collected studies using the same questionnaire (Table 2). Duplicates will be removed at this stage. We will obtain full reports for all the titles that appear to meet the inclusion criteria or where there is any uncertainty, and these will be reviewed according to the matrix method (26). First, we will read through the abstract, and then review the study to determine if it corresponds to the inclusion and exclusion criteria. If so, a copy of the article will be downloaded and sorted in a folder. Studies not meeting the inclusion criteria will be removed, and the reasons for excluding studies will be recorded. We will seek additional information from the study authors when necessary to resolve questions about eligibility. Any disagreement between the authors will be solved by discussion or by a third author. Neither of the review authors will be blind to the journal titles, study authors or institutions.

For peer review only

For peer review only - http://bmjopen.bmj.com/site/about/guidelines.xhtml

Table 2 Screening and extraction form	
**Level 1: Title and abstract review**	
1.Is this title written in English or Swedish?	Yes/no
2.Does it seem to address AN in general psychiatric inpatient care?	Yes/no
**Level 2: Full-text review**	
1.Is there any reason this article should be excluded?	
If yes:<br>What is the reason for excluding?	1. Not in English/Swedish<br>2. Not about AN in general psychiatric inpatient care<br>3. No full text available despite efforts to retrieve
If no:<br>What is the bibliographical information?	1. Author/s<br>2. Title<br>3. Country<br>4. Publication year
2. What type of publications?	a. Theoretical (commentary/viewpoint/letter)<br>b. Empirical (qualitative/quantitative/case report)

For peer review only

Data extraction, analysis and synthesis

Data will be analysed using a constant comparison method as described by Whittemore and Knafl's (20). The analysis is performed in four steps: 1) data reduction, 2) data overview, 3) data comparison and conclusions, and 4) verification of data. In the initially step of the analysis, the selected articles are read repeatedly for an overall picture of these content. Based on affiliation regarding focus and content, the articles are then divided into different groups. Answers to the study`s research questions are marked with a colour and coded with a number, making it easier to trace the data back to the primary sources. In the second step, the answers are sorted into preliminary subcategories and compiled into a matrix (a data overview) for a better overview of the analysed data. In the third step, new patterns and relationships are identified both within and outside primary data sources, which leads to new subcategories. In the fourth step of the analysis, the data are summarized and verified in accordance with Whittemore and Knafl (20).

When using this method, the goal of the data analysis is a thorough and impartial interpretation of the primary source that leads to new understanding and broader knowledge in the area studied. We will also aim to investigate whether a gender and power perspective was used in the studies. Discrepancies will be solved through discussion, and we will contact the study authors to resolve any uncertainties. Data extraction will be carried out by the first author and verified by the other authors in order to reduce bias and reduce errors in data extraction. In line with Whittemore and Knafl (20), we will provide a table summarizing all published studies included in the review, comparing their design, population, reference standard, dataset size, data processing methods, and diagnostic accuracy. Using this method, we will systematically summarize the narrative and present the synthesis in text and tables to explain the findings of the studies. By doing this, the findings will be explored and summarized both within and between the studies.

For peer review only - http://bmjopen.bmj.com/site/about/guidelines.xhtml

Participatory design

To broaden the creation of knowledge and to create space for the patient perspective we will involve persons with their own experiences of general psychiatric inpatient care for AN in the research process. Patients have a right to have an input in research, as this can reduce power imbalances, bring a lived-experience perspective, and increase relevance and transparency in research (27). The level of involvement will be as co-researchers.

Competing interest statement

None to declare.

DISCUSSION

This review will summarize the content of care for persons with AN in general psychiatric inpatient care. By doing this we will identify research gaps that can inform further research on how to best create opportunities for personal recovery for these patients.

A strength of this study is that it includes all kinds of care (nursing, psychological, medicine, etc.) for persons with AN in this context. This will give an overall picture of the content of care and reduce bias in interpretation of different types of care. One limitation is that we might miss articles due to our search strategy – we will only use three data bases, and there might be relevant articles in a data base that we did not search. Moreover, articles might be missed as some key search terms might not be included in their title or abstract. Despite this, this study will highlight what general psychiatric inpatient care has to offer persons with AN today. The results from this review will provide insight into an under-researched area.

For peer review only

REFERENCES

1. van Eeden AE, van Hoeken D, Hoek HW. Incidence, prevalence and mortality of anorexia nervosa and bulimia nervosa. Curr Opin Psychiatry. 2021;34(6):515–24.

2. Robinson PH, Kukucska R, Guidetti G, Leavey G. Severe and enduring anorexia nervosa (SEED-AN): a qualitative study of patients with 20+ years of anorexia nervosa. Eur Eat Disord Rev J Eat Disord Assoc. 2015;23(4):318–26.

3. Hannon J, Eunson L, Munro C. The patient experience of illness, treatment, and change, during intensive community treatment for severe anorexia nervosa. Eat Disord. 2017;25(4):279–96.

4. Harrison A, Mountford VA, Tchanturia K. Social anhedonia and work and social functioning in the acute and recovered phases of eating disorders. Psychiatry Res. 2014;218(1–2):187–94.

5. Erikson MG, Hansson B, Lundblad S. The possible selves of adult women with anorexia nervosa. Eat Disord. 2012;20(4):288–99.

6. Nordbø RHS, Espeset EMS, Gulliksen KS, Skårderud F, Holte A. The meaning of self-starvation: qualitative study of patients' perception of anorexia nervosa. Int J Eat Disord. 2006;39(7):556–64.

7. Tully SM, Bucci S, Berry K. 'My life isn't my life, it's the systems': a qualitative exploration of women's experiences of day-to-day restrictive practices as inpatients. J Psychiatr Ment Health Nurs. 2023;30(1):110–22.

8. Lindgren BM, Aminoff C, Hällgren Graneheim U. Features of everyday life in psychiatric inpatient care for self-harming: an observational study of six women. Issues Ment Health Nurs. 2015;36(2):82–8.

9. Salberg J, Bäckström J, Röing M, Öster C. Ways of understanding nursing in psychiatric inpatient care - a phenomenographic study. J Nurs Manag. 2019;27(8):1826–34.

10. Schmidt M, Stjernswärd S, Garmy P, Janlöv AC. Encounters with persons who frequently use psychiatric emergency services: healthcare professionals' views. Int J Environ Res Public Health. 2020;17(3):1012.

11. Matsuoka S. Recovery-oriented nursing care based on cultural sensitivity in community psychiatric nursing. Int J Ment Health Nurs. 2021;30(2):563–73.

12. Zugai JS, Stein-Parbury J, Roche M. Therapeutic alliance, anorexia nervosa and the inpatient setting: a mixed methods study. J Adv Nurs. 2018;74(2):443–53.

13. Sibeoni J, Orri M, Colin S, Valentin M, Pradère J, Revah-Levy A. The lived experience of anorexia nervosa in adolescence, comparison of the points of view of adolescents, parents, and professionals: a metasynthesis. Int J Nurs Stud. 2017;65:25–34.

14. Davidson AR, Braham S, Dasey L, Reidlinger DP. Physicians' perspectives on the treatment of patients with eating disorders in the acute setting. J Eat Disord. 2019;7:1.

15. National Institute for Health and Care Excellence. Eating disorders: recognition and treatment [Internet]. NICE; 2020. Available from: https://www.nice.org.uk/guidance/ng69/chapter/Recommendations#treating-anorexia-nervosa

For peer review only - http://bmjopen.bmj.com/site/about/guidelines.xhtml

16. World Health Organization. Integrated people-centred care [Internet]. World Health Organization; 2016. Available from: https://www.who.int/health-topics/integrated-people-centered-care#tab=tab_1

17. Williams R, Smith M, Wright D. Anorexia: a literature review of young people's experiences of hospital treatment. Nurs Child Young People. 2021 Mar 4;33(2):10–7.

18. Wetzler S, Hackmann C, Peryer G, Clayman K, Friedman D, Saffran K, et al. A framework to conceptualize personal recovery from eating disorders: a systematic review and qualitative meta-synthesis of perspectives from individuals with lived experience. Int J Eat Disord. 2020;53(8):1188–203.

19. Statens Beredning för Medicinsk och Social Utvärdering. Ätstörningar - en sammanställning av systematiska översikter av kvalitativ forskning utifrån patientens, närståendes och hälso- och sjukvårdens perspektiv [Internet]. SBU; 2019. Available from: https://www.sbu.se/sv/publikationer/sbu-bereder/atstorningar/?lang=sv

20. Whittemore R, Knafl K. The integrative review: updated methodology. J Adv Nurs. 2005;52(5):546–53.

21. Polit DF, Beck CT. Nursing research: generating and assessing evidence for nursing practice. Eleventh edition, international edition. Philadelphia Baltimore New York: Wolters Kluwer; 2021.

22. Lindsey WT, Olin BR. PubMed searches: overview and strategies for clinicians. Nutr Clin Pract. 2013;28(2):165–76.

23. Page MJ, McKenzie JE, Bossuyt PM, Boutron I, Hoffmann TC, Mulrow CD, et al. The PRISMA 2020 statement: an updated guideline for reporting systematic reviews. BMJ. 2021;372:n71.

24. Ouzzani M, Hammady H, Fedorowicz Z, Elmagarmid A. Rayyan - a web and mobile app for systematic reviews. Syst Rev. 2016;5(1):210.

25. Hong QN, Fàbregues S, Bartlett G, Boardman F, Cargo M, Dagenais P, et al. The Mixed Methods Appraisal Tool (MMAT) version 2018 for information professionals and researchers. Educ Inf. 2018;34(4):285–91.

26. Garrard J. Health sciences literature review made easy: the matrix method, sixth edition. Sixth. Jones & Barlett Learning; 2021.

27. Greenhalgh T, Hinton L, Finlay T, Macfarlane A, Fahy N, Clyde B, et al. Frameworks for supporting patient and public involvement in research: systematic review and co-design pilot. Health Expect. 2019;22(4):785–801.

For peer review only - http://bmjopen.bmj.com/site/about/guidelines.xhtml

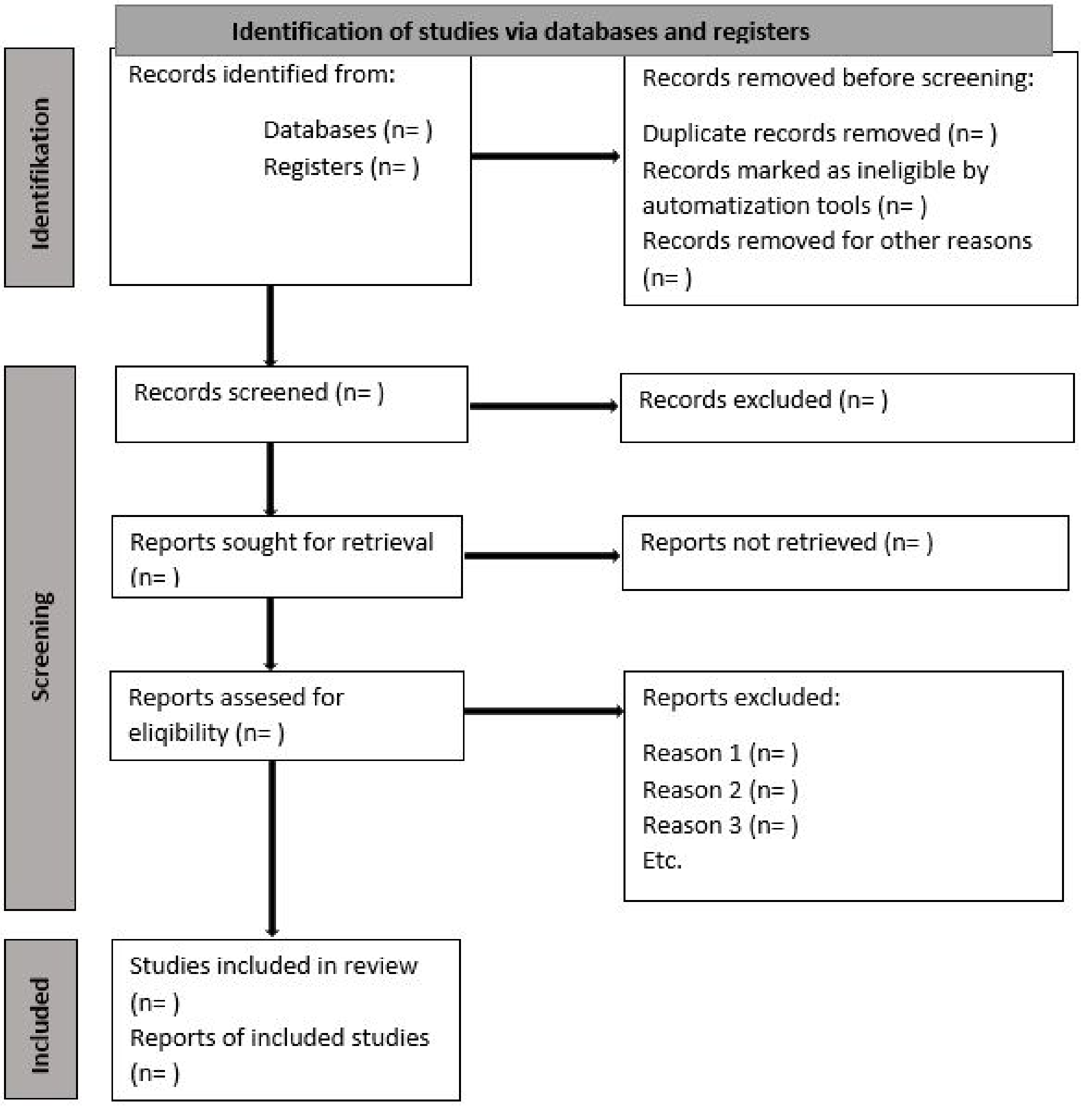
Identification of studies via databases and registers
Identifikation
Screening
Included
Records identified from:
Databases (n=)
Registers (n=)
Records removed before screening:
Duplicate records removed (n=)
Records marked as ineligible by automatization tools (n=)
Records removed for other reasons (n=)
Records screened (n=)
Records excluded (n=)
Reports sought for retrieval (n=)
Reports not retrieved (n=)
Reports assesed for eligibility (n=)
Reports excluded:
Reason 1 (n=)
Reason 2 (n=)
Reason 3 (n=)
Etc.
Studies included in review (n=)
Reports of included studies (n=)

y only

PRISMA-P (Preferred Reporting Items for Systematic review and Meta-Analysis Protocols) 2015 checklist: recommended items to address in a systematic review protocol*

Section and topic	Item No	Checklist item
**ADMINISTRATIVE INFORMATION**		
Title:		
Identification	1a	Identify the report as a protocol of a systematic review
Update	1b	If the protocol is for an update of a previous systematic review, identify as such
Registration	2	If registered, provide the name of the registry (such as PROSPERO) and registration number
Authors:		
Contact	3a	Provide name, institutional affiliation, e-mail address of all protocol authors; provide physical mailing address of corresponding author
Contributions	3b	Describe contributions of protocol authors and identify the guarantor of the review
Amendments	4	If the protocol represents an amendment of a previously completed or published protocol, identify as such and list changes; otherwise, state plan for documenting important protocol amendments
Support:		
Sources	5a	Indicate sources of financial or other support for the review
Sponsor	5b	Provide name for the review funder and/or sponsor
Role of sponsor or funder	5c	Describe roles of funder(s), sponsor(s), and/or institution(s), if any, in developing the protocol
**INTRODUCTION**		
Rationale	6	Describe the rationale for the review in the context of what is already known
Objectives	7	Provide an explicit statement of the question(s) the review will address with reference to participants, interventions, comparators, and outcomes (PICO)
**METHODS**		
Eligibility criteria	8	Specify the study characteristics (such as PICO, study design, setting, time frame) and report characteristics (such as years considered, language, publication status) to be used as criteria for eligibility for the review
Information sources	9	Describe all intended information sources (such as electronic databases, contact with study authors, trial registers or other grey literature sources) with planned dates of coverage
Search strategy	10	Present draft of search strategy to be used for at least one electronic database, including planned limits, such that it could be repeated
Study records:		
Data management	11a	Describe the mechanism(s) that will be used to manage records and data throughout the review

For peer review only

Selection process	11b	State the process that will be used for selecting studies (such as two independent reviewers) through each phase of the review (that is, screening, eligibility and inclusion in meta-analysis)
Data collection process	11c	Describe planned method of extracting data from reports (such as piloting forms, done independently, in duplicate), any processes for obtaining and confirming data from investigators
Data items	12	List and define all variables for which data will be sought (such as PICO items, funding sources), any pre-planned data assumptions and simplifications
Outcomes and prioritization	13	List and define all outcomes for which data will be sought, including prioritization of main and additional outcomes, with rationale
Risk of bias in individual studies	14	Describe anticipated methods for assessing risk of bias of individual studies, including whether this will be done at the outcome or study level, or both; state how this information will be used in data synthesis
Data synthesis	15a	Describe criteria under which study data will be quantitatively synthesised
	15b	If data are appropriate for quantitative synthesis, describe planned summary measures, methods of handling data and methods of combining data from studies, including any planned exploration of consistency (such as I^2, Kendall's τ)
	15c	Describe any proposed additional analyses (such as sensitivity or subgroup analyses, meta-regression)
	15d	If quantitative synthesis is not appropriate, describe the type of summary planned
Meta-bias(es)	16	Specify any planned assessment of meta-bias(es) (such as publication bias across studies, selective reporting within studies)
Confidence in cumulative evidence	17	Describe how the strength of the body of evidence will be assessed (such as GRADE)

*** It is strongly recommended that this checklist be read in conjunction with the PRISMA-P Explanation and Elaboration (cite when available) for important clarification on the items. Amendments to a review protocol should be tracked and dated. The copyright for PRISMA-P (including checklist) is held by the PRISMA-P Group and is distributed under a Creative Commons Attribution Licence 4.0.**

From: Shamseer L, Moher D, Clarke M, Ghersi D, Liberati A, Petticrew M, Shekelle P, Stewart L, PRISMA-P Group. Preferred reporting items for systematic review and meta-analysis protocols (PRISMA-P) 2015: elaboration and explanation. BMJ. 2015 Jan 2;349(jan02 1):g7647.

For peer review only

BMJ Open

General psychiatric inpatient care for persons with anorexia nervosa - an integrative literature review protocol

Journal:	*BMJ Open*
Manuscript ID	bmjopen-2023-076437.R1
Article Type:	Protocol
Date Submitted by the Author:	30-Aug-2023
Complete List of Authors:	Sandsten, Anna; Umea universitet Medicinska fakulteten, Nursing<br>Lindgren, Britt-Marie; Umeå universitet Medicinska fakulteten, Nursing<br>Strömbäck, Maria; Umea University Department of Community Medicine and Rehabilitation, Nursing<br>Ejneborn-Looi, Git-Marie; Luleå University of Technology, Department of Health Sciences<br>Gabrielsson, Sebastian; Luleå University of Technology, Department of Health Sciences
**Primary Subject Heading**:	Mental health
Secondary Subject Heading:	Mental health
Keywords:	Eating disorders < PSYCHIATRY, Hospitalization, PSYCHIATRY, MENTAL HEALTH

SCHOLARONE™
Manuscripts

General psychiatric inpatient care for persons with anorexia nervosa – an integrative literature review protocol

Anna Sandsten, PhD Student, MSc, Specialist Nurse in Psychiatric Care, Department of Nursing, Umeå Center for Gender Studies, Gender Research School, Umeå University. E-mail: anna.sandsten@umu.se

Britt-Marie Lindgren, PhD, Associated Professor, Specialist Nurse in Psychiatric Care, Department of Nursing, Umeå University. E-mail: britt-marie.lindgren@umu.se. Orcid nr 0000-0002-3360 5589

Maria Strömbäck, PhD, Senior Lecturer, Specialist Physiotherapist, Department of Community Medicine and Rehabilitation, Umeå University. E-mail: maria.stromback@umu.se

Git-Marie Ejneborn-Looi, PhD, Senior Lecturer, Specialist Nurse in Psychiatric Care, Department of Health, Learning and Technology, Luleå University of Technology. E-mail: git-marie.ejneborn-looi@ltu.se

Sebastian Gabrielsson, PhD, Associate Professor, Specialist Nurse in Psychiatric Care, Department of Health, Learning and Technology, Luleå University of Technology. E-mail: sebastian.gabrielsson@ltu.se Orcid nr 0000-0002-1624.1795

Corresponding author Anna Sandsten, e-mail: anna.sandsten@gmail.com, +46702349993.

Author contributions All authors contributed to the conception of the review protocol and the study design of the review. Anna Sandsten (AS) and Sebastian Gabrielsson (SG) developed the search strategy together with a librarian and prepared supplementary materials. AS lead the writing of the manuscript, incorporated feedback from SG, Git-Marie Ejneborn-Looi (GME), Britt-Marie Lindgren (BML) and Maria Strömbäck (MS) through multiple drafts. AS and SG prepared correspondence with the publisher. AS, SG, GME, BML and MS contributed to the final manuscript and agreed with its publication.

ABSTRACT

Introduction People diagnosed with anorexia nervosa are offered or compelled to undergo various treatments and therapies. In Sweden, the lack of highly specialized eating disorder services means that people with anorexia nervosa might be cared for in general psychiatric wards. However, there has been limited research attention paid to how this specific context might benefit or harm persons with anorexia nervosa. There is also limited evidence regarding which form of care is most effective in anorexia nervosa and which type of treatment is optimal in general psychiatric inpatient care. Our intention is to highlight personal and professional perspectives of care for patients with anorexia nervosa in this context, with a focus on person-centred care, gender, and power. This protocol describes an integrative literature review aiming to synthesize existing knowledge on general psychiatric inpatient care for persons with anorexia nervosa.

Methods and analysis The study will be an integrative literature review following Whittemore and Knafl. We will search the academic databases PubMed, CINAHL, and PsycInfo in accordance with a specific search strategy determined in collaboration with a librarian with expertise on systematic reviews. Data will be analysed using a constant comparison method. Persons with their own experience of anorexia nervosa in general psychiatric inpatient care will be involved as co-researchers.

Ethics and dissemination As this is a literature review, formal ethical review is not required. The findings will be disseminated through peer-reviewed publication and presented at conferences related to this field.

Amendments If we need to amend this protocol, we will give the date of each amendment, describe the change, and give the rationale in this section.

Sponsor and founding This research received no specific grant from any funding agency in the public, commercial, or non-profit sectors.

For peer review only - http://bmjopen.bmj.com/site/about/guidelines.xhtml

PROSPERO registration number CRD42023426095

STRENGTHS AND LIMITATIONS OF THIS STUDY

- This review will provide knowledge of the content of nursing in general psychiatric inpatient care for persons with anorexia nervosa.
- Persons with their own experiences of anorexia nervosa in general inpatient care will contribute their knowledge and experience as co-researchers and through reading and commenting on the article.
- The publication of this protocol ensures transparency, and evaluation by independent reviewers of all studies during screening and selection reduces the risk of bias in the selection of included studies.
- An integrative literature review combines findings from quantitative and qualitative research which ensures full coverage of research knowledge in the field.
- A limitation is that we exclude studies in languages other than Swedish and English which may lead to potentially relevant studies not being included.

INTRODUCTION

Anorexia nervosa (AN) is reported worldwide among people of all genders and ages. The incidence among younger persons (aged <15) has increased in recent decades, but the reason for this remains unclear and requires further research (1). Quality of life for those having AN can be very low, and severe long-term AN significantly increases the risk of premature death due to physical stress or suicide (2). The psychological foundation of AN can be described by feelings of self-criticism, low self-esteem, isolation, captivity in the disease, and hopelessness about the possibility of a good life (3). On the one hand, AN is a psychiatric diagnosis with a large negative impact on social ability (4), but on the other, it can be experienced as psychologically meaningful. For example, strict rules regarding diet and activity can create structure and predictability in everyday life. Furthermore, AN can function as a strategy to avoid difficult feelings, and can contribute to the experience of control and inner strength (5,6).

Persons who can no longer manage to cope with their psychiatric illness at home are able to receive psychiatric inpatient care at a hospital (7). However, the lack of highly specialized eating disorder services means that persons with AN might be cared for in general psychiatric wards, which constitute an environment characterized by rules, locked doors, and a lack of choice (8). This can be stressful, but at the same time being in a hospital can allow feelings of being safe. General psychiatric inpatient care can be voluntary or coercive, and persons experiencing AN can be offered or compelled to undergo various treatments and therapies (7).

The organization of psychiatric services differs worldwide. Inpatient care may be provided in large mental health institutions or in community or general hospital settings. For the purpose of this review, we understand general psychiatric inpatient care to encompass any 24-hour service providing psychiatric care and residence for people with various psychiatric conditions. General psychiatric inpatient care in psychiatry typically means that persons with different diagnoses are cared for in the same place. This leads to a mix of persons with different needs (8) - for example, a person experiencing a manic episode may need reduced stimuli at the same time as a person with depression may need support in finding meaningful activities, and a person with AN may need support in reducing their activity level. Staff describe difficulties in switching between patients' different needs (9), and wish for strategies

For peer review only - http://bmjopen.bmj.com/site/about/guidelines.xhtml

and resources for a meaningful care (10). General psychiatric inpatient care can be understood as a closed system which has specific ideas and expectations of both staff and patients (11). Staff describe difficulties in maintaining relationships with persons with AN who experience positive aspects of the disease, leading to ambivalent feelings towards care (12,13). Perceiving a person as "difficult to treat" can inspire feelings of insecurity in staff. Uncertainty may lead to an increased tendency to rely on guidelines in the care of persons with AN, rather than finding individualized solutions (14).

According to the National Institute for Health and Care Excellence (15), treatment for AN should focus on psychoeducation, monitoring of weight, mental and physical health, and risk factors. Care should also be multidisciplinary and involve family members. These recommendations highlight that helping persons with AN to reach a healthy body weight is a key goal, and that weight gain is key in supporting other psychological, physical, and quality of life changes that are needed for improvement or recovery. In person-centred care, it is important to empower the patient to have an active role in their own care, to create good relationships, and to see the whole person beyond the disease (16). In recovery-oriented care, the patient owns their narratives in order to create security and enable them to use dialogue to express their expectations and wishes regarding the care and the goals of the care (11). To enable a turning point towards recovery for persons with AN, it is of great importance for the care to focus on both physical and psychological well-being (17). Personal recovery from AN is characterized by being seen and understood as an individual, by receiving support in finding meaning in life beyond illness, by feeling hope for recovery, and by regaining control and assuming responsibility over life (18).

Research indicates that care is influenced by societal gender norms, which in turn affect how healthcare professionals interact with patients and interpret patient complaints. Consequently, these gendered expectations may result in patients receiving varying examinations, treatments, and medications without sufficient medical justification (19). This study will summarize the care provided to individuals with AN within the setting of general psychiatric inpatient care. Emphasis will be placed on examining the content of care and the outcomes of care, exploring the discourse surrounding the diagnosis, and of the persons having the diagnose. Additionally, the study will shed light on gendered expectations and power imbalances that exist in this particular context.

Aim

The aim of this study is to synthesize existing knowledge on general psychiatric inpatient care for persons with AN.

For peer review only - http://bmjopen.bmj.com/site/about/guidelines.xhtml

Research questions

The review will seek to answer the following research questions:

- How is the content of care described?
- What is the focus of these descriptions? Opportunities/limitations?
- What outcomes are described?
- How are persons experiencing AN described?
- How is AN described?
- Which factors influence care for persons with AN?
- Are gender or power perspectives included in studies?
- How is person-centred care described?

METHODS AND ANALYSIS

Study design

This will be an integrative literature review conducted according to the method of Whittemore and Knafl´s (20). The purpose of using this method is to cover a large number of studies and to avoid excluding any research on the basis of method.

Inclusion and exclusion criteria

The studies included in the review will be restricted to those where the setting is general psychiatric inpatient care. Studies describing general psychiatric care for persons of all ages with AN will be included. We will also include studies that describe interventions in general psychiatric inpatient care for persons with AN, and that have a patient, relative, and/or staff perspective. Only studies that are peer-reviewed and published in scientific journals will be included. Language of publication will be restricted to English and Swedish, due to the authors` knowledge of languages. Studies with a focus on eating disorders in general will be excluded.

Information sources

The literature search will start in the spring of 2023, and we will aim for the study to be finished in January of 2024. Based on the specific purpose of the study and its inclusion and exclusion criteria, we will systematically search for knowledge and understanding in studies published in academic databases (20,21). We will use medical subject headings (MeSH) (22), CINAHL headings, and text words related to AN and inpatient care. Databases used in the search will be PubMed, CINAHL, and PsycINFO. We will also hand-search the reference lists of research and review articles to make sure that all relevant material has been captured.

Search strategy

The key concepts in this study are anorexia nervosa and inpatient care, and so the search terms will be "anorexia nervosa", "anorexia", "inpatients", "inpatient care", and "hospitalization". These search terms are combined using the Boolean operators AND and OR (22). A block search will be performed with search terms in the form of MeSH-terms (22),

For peer review only - http://bmjopen.bmj.com/site/about/guidelines.xhtml

For peer review only

CINAHL headings, and free text. The literature search will be limited to peer-reviewed articles published in English. No study design or time limitations will be imposed on the search, and similar searches will be performed in all databases (Table 1). Any duplicates will be removed. A PRISMA flow diagram (Figure 1) will be used to organize and summarize the search process (23). Finally, we will circulate a bibliography of the included articles to the systematic review team. The literature search strategy has been developed in collaboration with a librarian with expertise in systematic reviews at Luleå University of Technology.

**Table 1**	Draft PubMed search		
**Database: PubMed Date: 03-07-23**	Search terms	Limitations	Results
**#1**	anorexia nervosa [MeSH Terms] OR anorexia [Title/Abstract]		36 701
**#2**	inpatients [MeSH Terms] OR hospitalization [MeSH Terms] OR inpatient care [Title/Abstract]		312 995
**#3**	#1 AND #2	Full text, English, Swedish	991

Data management and quality appraisal

The literature search results will be uploaded to Rayyan, an Internet-based software program that can be used to facilitate collaboration among reviewers during the process of selecting studies (24). To appraise the methodological quality of studies with different types of methods (i.e. qualitative, quantitative, and mixed-methods studies), the Mixed Methods Appraisal Tool (MMAT) (25) will be used. The MMAT guides users through four steps: 1) the screening questions are applied to all studies. 2) for each relevant study, the type of design is determined and the corresponding criteria are used to appraise the study's quality, 3) two independent reviewers conduct the appraisal process and 4) an overall quality score is determined for each study (25). Because of the aim of this review, no studies will be excluded due to quality instead quality will be visualized and reflected on in the analysis section.

Selection process

Two authors will independently examine the collected studies using the same questionnaire (Table 2). Duplicates will be removed at this stage. We will obtain full reports for all the titles that appear to meet the inclusion criteria or where there is any uncertainty, and these will be reviewed according to the matrix method (26). First, we will read through the abstract, and then review the study to determine if it corresponds to the inclusion and exclusion criteria. If so, a copy of the article will be downloaded and sorted in a folder. Studies not meeting the inclusion criteria will be removed, and the reasons for excluding studies will be recorded. We will seek additional information from the study authors when necessary to resolve questions about eligibility. Any disagreement between the authors will be solved by discussion or by a

For peer review only - http://bmjopen.bmj.com/site/about/guidelines.xhtml

third author. Neither of the review authors will be blind to the journal titles, study authors or institutions.

Table 2 Screening and extraction form

**Level 1: Title and abstract review**	
1.Is this title written in English or Swedish?	Yes/no
2.Does it seem to address AN in general psychiatric inpatient care?	Yes/no
**Level 2: Full-text review**	
1.Is there any reason this article should be excluded?	
If yes:<br>What is the reason for excluding?	1. Not in English/Swedish<br>2. Not about AN in general psychiatric inpatient care<br>3. No full text available despite efforts to retrieve
If no:<br>What is the bibliographical information?	1. Author/s<br>2. Title<br>3. Country<br>4. Publication year
2. What type of publications?	a. Theoretical (commentary/viewpoint/letter)<br>b. Empirical (qualitative/quantitative/case report)

For peer review only

Data extraction, analysis and synthesis

Data will be analysed using a constant comparison method as described by Whittemore and Knafl's (20). The analysis is performed in four steps: 1) data reduction, 2) data overview, 3) data comparison and conclusions, and 4) verification of data. In the initially step of the analysis, the selected articles are read repeatedly for an overall picture of these content. Based on affiliation regarding focus and content, the articles are then divided into different groups. Answers to the study`s research questions are marked with a colour and coded with a number, making it easier to trace the data back to the primary sources. In the second step, the answers are sorted into preliminary subcategories and compiled into a matrix (a data overview) for a better overview of the analysed data. In the third step, new patterns and relationships are identified both within and outside primary data sources, which leads to new subcategories. In the fourth step of the analysis, the data are summarized and verified in accordance with Whittemore and Knafl (20).

When using this method, the goal of the data analysis is a thorough and impartial interpretation of the primary source that leads to new understanding and broader knowledge in the area studied. We will also aim to investigate whether a gender and power perspective was used in the studies. Discrepancies will be solved through discussion, and we will contact the study authors to resolve any uncertainties. Data extraction will be carried out by the first author and verified by the other authors in order to reduce bias and reduce errors in data extraction. In line with Whittemore and Knafl (20), we will provide a table summarizing all published studies included in the review, comparing their design, population, reference standard, dataset size, data processing methods, and diagnostic accuracy. Using this method,

For peer review only - http://bmjopen.bmj.com/site/about/guidelines.xhtml

we will systematically summarize the narrative and present the synthesis in text and tables to explain the findings of the studies. By doing this, the findings will be explored and summarized both within and between the studies.

Patient and public involvement

Patients have a right to have an input in research, as this can reduce power imbalances, bring a lived-experience perspective, and increase relevance and transparency in research (27). To broaden the creation of knowledge and to create space for the patient perspective we will involve a person with own experiences of general psychiatric inpatient care for AN in the research process. The level of involvement will be as co-researcher in study selection, quality appraisal, data extraction, analysis and synthesis.

Competing interest statement

None to declare.

DISCUSSION

This review will summarize the content of care for persons with AN in general psychiatric inpatient care. By doing this we will identify research gaps that can inform further research on how to best create opportunities for personal recovery for these patients.

A strength of this study is that it includes all kinds of care (nursing, psychological, medicine, etc.) for persons with AN in this context. This will give an overall picture of the content of care and reduce bias in interpretation of different types of care. One limitation is that we might miss articles due to our search strategy – we will only use three data bases, and there might be relevant articles in a data base that we did not search. Moreover, articles might be missed as some key search terms might not be included in their title or abstract. Despite this, this study will highlight what general psychiatric inpatient care has to offer persons with AN today. The results from this review will provide insight into an under-researched area.

For peer review only

For peer review only - http://bmjopen.bmj.com/site/about/guidelines.xhtml

REFERENCES

1. van Eeden AE, van Hoeken D, Hoek HW. Incidence, prevalence and mortality of anorexia nervosa and bulimia nervosa. Curr Opin Psychiatry. 2021;34(6):515–24.

2. Robinson PH, Kukucska R, Guidetti G, Leavey G. Severe and enduring anorexia nervosa (SEED-AN): a qualitative study of patients with 20+ years of anorexia nervosa. Eur Eat Disord Rev J Eat Disord Assoc. 2015;23(4):318–26.

3. Hannon J, Eunson L, Munro C. The patient experience of illness, treatment, and change, during intensive community treatment for severe anorexia nervosa. Eat Disord. 2017;25(4):279–96.

4. Harrison A, Mountford VA, Tchanturia K. Social anhedonia and work and social functioning in the acute and recovered phases of eating disorders. Psychiatry Res. 2014;218(1–2):187–94.

5. Erikson MG, Hansson B, Lundblad S. The possible selves of adult women with anorexia nervosa. Eat Disord. 2012;20(4):288–99.

6. Nordbø RHS, Espeset EMS, Gulliksen KS, Skårderud F, Holte A. The meaning of self-starvation: qualitative study of patients' perception of anorexia nervosa. Int J Eat Disord. 2006;39(7):556–64.

7. Tully SM, Bucci S, Berry K. 'My life isn't my life, it's the systems': a qualitative exploration of women's experiences of day-to-day restrictive practices as inpatients. J Psychiatr Ment Health Nurs. 2023;30(1):110–22.

8. Lindgren BM, Aminoff C, Hällgren Graneheim U. Features of everyday life in psychiatric inpatient care for self-harming: an observational study of six women. Issues Ment Health Nurs. 2015;36(2):82–8.

9. Salberg J, Bäckström J, Röing M, Öster C. Ways of understanding nursing in psychiatric inpatient care - a phenomenographic study. J Nurs Manag. 2019;27(8):1826–34.

10. Schmidt M, Stjernswärd S, Garmy P, Janlöv AC. Encounters with persons who frequently use psychiatric emergency services: healthcare professionals' views. Int J Environ Res Public Health. 2020;17(3):1012.

11. Matsuoka S. Recovery-oriented nursing care based on cultural sensitivity in community psychiatric nursing. Int J Ment Health Nurs. 2021;30(2):563–73.

12. Zugai JS, Stein-Parbury J, Roche M. Therapeutic alliance, anorexia nervosa and the inpatient setting: a mixed methods study. J Adv Nurs. 2018;74(2):443–53.

13. Sibeoni J, Orri M, Colin S, Valentin M, Pradère J, Revah-Levy A. The lived experience of anorexia nervosa in adolescence, comparison of the points of view of adolescents, parents, and professionals: a metasynthesis. Int J Nurs Stud. 2017;65:25–34.

14. Davidson AR, Braham S, Dasey L, Reidlinger DP. Physicians' perspectives on the treatment of patients with eating disorders in the acute setting. J Eat Disord. 2019;7:1.

15. National Institute for Health and Care Excellence. Eating disorders: recognition and treatment [Internet]. NICE; 2020. Available from: https://www.nice.org.uk/guidance/ng69/chapter/Recommendations#treating-anorexia-nervosa

For peer review only - http://bmjopen.bmj.com/site/about/guidelines.xhtml

16. World Health Organization. Integrated people-centred care [Internet]. World Health Organization; 2016. Available from: https://www.who.int/health-topics/integrated-people-centered-care#tab=tab_1

17. Williams R, Smith M, Wright D. Anorexia: a literature review of young people's experiences of hospital treatment. Nurs Child Young People. 2021 Mar 4;33(2):10–7.

18. Wetzler S, Hackmann C, Peryer G, Clayman K, Friedman D, Saffran K, et al. A framework to conceptualize personal recovery from eating disorders: a systematic review and qualitative meta-synthesis of perspectives from individuals with lived experience. Int J Eat Disord. 2020;53(8):1188–203.

19. Statens Beredning för Medicinsk och Social Utvärdering. Ätstörningar - en sammanställning av systematiska översikter av kvalitativ forskning utifrån patientens, närståendes och hälso- och sjukvårdens perspektiv [Internet]. SBU; 2019. Available from: https://www.sbu.se/sv/publikationer/sbu-bereder/atstorningar/?lang=sv

20. Whittemore R, Knafl K. The integrative review: updated methodology. J Adv Nurs. 2005;52(5):546–53.

21. Polit DF, Beck CT. Nursing research: generating and assessing evidence for nursing practice. Eleventh edition, international edition. Philadelphia Baltimore New York: Wolters Kluwer; 2021.

22. Lindsey WT, Olin BR. PubMed searches: overview and strategies for clinicians. Nutr Clin Pract. 2013;28(2):165–76.

23. Page MJ, McKenzie JE, Bossuyt PM, Boutron I, Hoffmann TC, Mulrow CD, et al. The PRISMA 2020 statement: an updated guideline for reporting systematic reviews. BMJ. 2021;372:n71.

24. Ouzzani M, Hammady H, Fedorowicz Z, Elmagarmid A. Rayyan - a web and mobile app for systematic reviews. Syst Rev. 2016;5(1):210.

25. Hong QN, Fàbregues S, Bartlett G, Boardman F, Cargo M, Dagenais P, et al. The Mixed Methods Appraisal Tool (MMAT) version 2018 for information professionals and researchers. Educ Inf. 2018;34(4):285–91.

26. Garrard J. Health sciences literature review made easy: the matrix method, sixth edition. Sixth. Jones & Barlett Learning; 2021.

27. Greenhalgh T, Hinton L, Finlay T, Macfarlane A, Fahy N, Clyde B, et al. Frameworks for supporting patient and public involvement in research: systematic review and co-design pilot. Health Expect. 2019;22(4):785–801.

For peer review only

For peer review only - http://bmjopen.bmj.com/site/about/guidelines.xhtml

Figure 1 Prisma flow diagram for systematic reviews

For peer review only

For peer review only - http://bmjopen.bmj.com/site/about/guidelines.xhtml

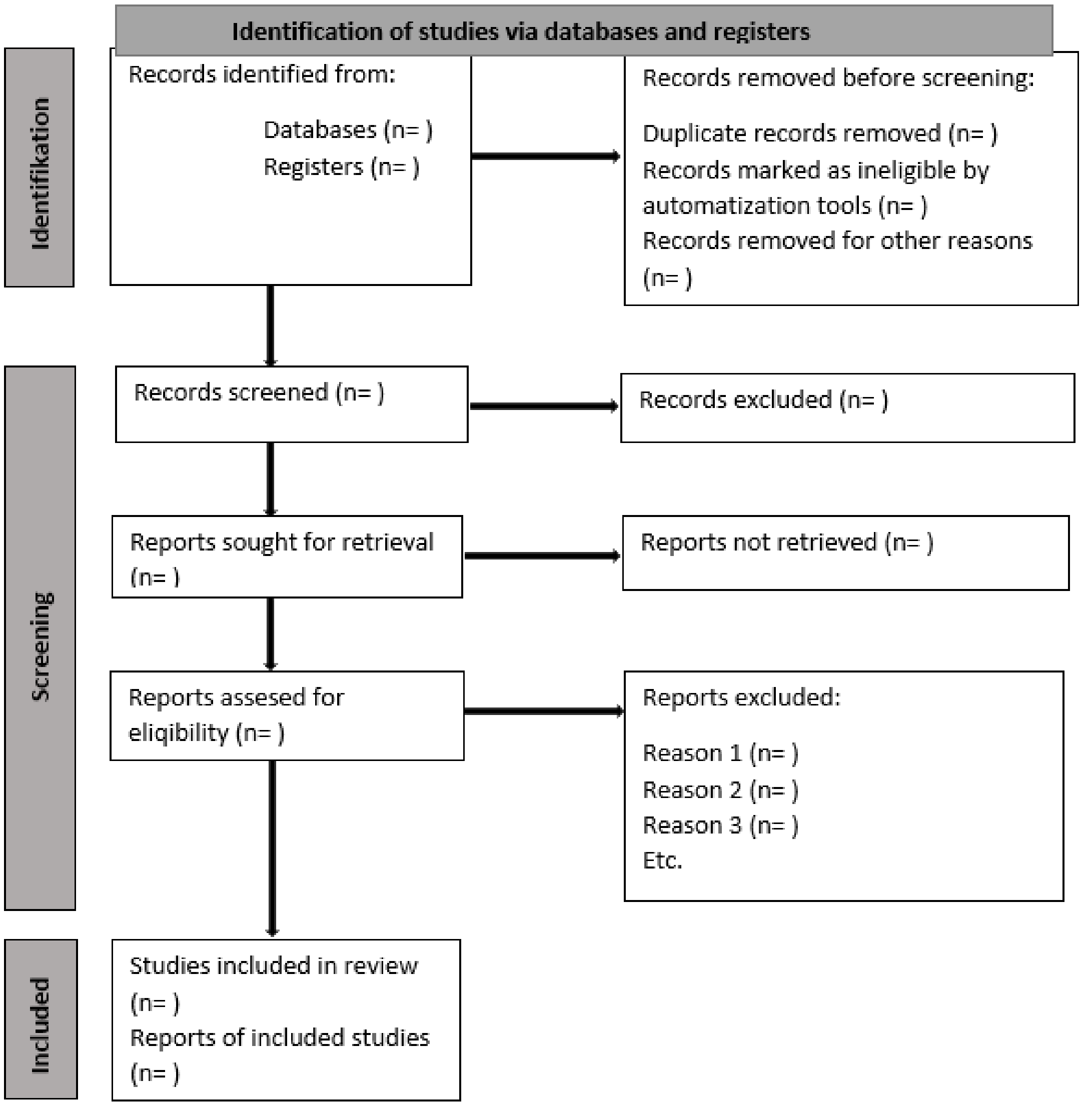
Identification of studies via databases and registers
Identifikation
Records identified from:
Databases (n=)
Registers (n=)
Records removed before screening:
Duplicate records removed (n=)
Records marked as ineligible by automatization tools (n=)
Records removed for other reasons (n=)
Screening
Records screened (n=)
Records excluded (n=)
Reports sought for retrieval (n=)
Reports not retrieved (n=)
Reports assesed for eliqibility (n=)
Reports excluded:
Reason 1 (n=)
Reason 2 (n=)
Reason 3 (n=)
Etc.
Included
Studies included in review (n=)
Reports of included studies (n=)

PRISMA-P (Preferred Reporting Items for Systematic review and Meta-Analysis Protocols) 2015 checklist: recommended items to address in a systematic review protocol*

Section and topic	Item No	Checklist item
**ADMINISTRATIVE INFORMATION**		
Title:		
Identification	1a	Identify the report as a protocol of a systematic review 1
Update	1b	If the protocol is for an update of a previous systematic review, identify as such na
Registration	2	If registered, provide the name of the registry (such as PROSPERO) and registration number 2
Authors:		
Contact	3a	Provide name, institutional affiliation, e-mail address of all protocol authors; provide physical mailing address of corresponding author 1
Contributions	3b	Describe contributions of protocol authors and identify the guarantor of the review 1
Amendments	4	If the protocol represents an amendment of a previously completed or published protocol, identify as such and list changes; otherwise, state plan for documenting important protocol amendments 1
Support:		
Sources	5a	Indicate sources of financial or other support for the review 1
Sponsor	5b	Provide name for the review funder and/or sponsor na
Role of sponsor or funder	5c	Describe roles of funder(s), sponsor(s), and/or institution(s), if any, in developing the protocol na
**INTRODUCTION**		
Rationale	6	Describe the rationale for the review in the context of what is already known 2,3
Objectives	7	Provide an explicit statement of the question(s) the review will address with reference to participants, interventions, comparators, and outcomes (PICO) 3
**METHODS**		
Eligibility criteria	8	Specify the study characteristics (such as PICO, study design, setting, time frame) and report characteristics (such as years considered, language, publication status) to be used as criteria for eligibility for the review 4
Information sources	9	Describe all intended information sources (such as electronic databases, contact with study authors, trial registers or other grey literature sources) with planned dates of coverage 4
Search strategy	10	Present draft of search strategy to be used for at least one electronic database, including planned limits, such that it could be repeated 4,5
Study records:		
Data management	11a	Describe the mechanism(s) that will be used to manage records and data throughout the review 5

For peer review only

Selection process	11b	State the process that will be used for selecting studies (such as two independent reviewers) through each phase of the review (that is, screening, eligibility and inclusion in meta-analysis)	5
Data collection process	11c	Describe planned method of extracting data from reports (such as piloting forms, done independently, in duplicate), any processes for obtaining and confirming data from investigators	6
Data items	12	List and define all variables for which data will be sought (such as PICO items, funding sources), any pre-planned data assumptions and simplifications	6
Outcomes and prioritization	13	List and define all outcomes for which data will be sought, including prioritization of main and additional outcomes, with rationale	na
Risk of bias in individual studies	14	Describe anticipated methods for assessing risk of bias of individual studies, including whether this will be done at the outcome or study level, or both; state how this information will be used in data synthesis	6
Data synthesis	15a	Describe criteria under which study data will be quantitatively synthesised	6
	15b	If data are appropriate for quantitative synthesis, describe planned summary measures, methods of handling data and methods of combining data from studies, including any planned exploration of consistency (such as I^2, Kendall's τ)	6
	15c	Describe any proposed additional analyses (such as sensitivity or subgroup analyses, meta-regression)	na
	15d	If quantitative synthesis is not appropriate, describe the type of summary planned	na
Meta-bias(es)	16	Specify any planned assessment of meta-bias(es) (such as publication bias across studies, selective reporting within studies)	6
Confidence in cumulative evidence	17	Describe how the strength of the body of evidence will be assessed (such as GRADE)	na

*** It is strongly recommended that this checklist be read in conjunction with the PRISMA-P Explanation and Elaboration (cite when available) for important clarification on the items. Amendments to a review protocol should be tracked and dated. The copyright for PRISMA-P (including checklist) is held by the PRISMA-P Group and is distributed under a Creative Commons Attribution Licence 4.0.**

From: Shamseer L, Moher D, Clarke M, Ghersi D, Liberati A, Petticrew M, Shekelle P, Stewart L, PRISMA-P Group. Preferred reporting items for systematic review and meta-analysis protocols (PRISMA-P) 2015: elaboration and explanation. BMJ. 2015 Jan 2;349(jan02 1):g7647.

For peer review only
